# Diaqua­bis[2-(4-bromo­phen­yl)acetato]bis­(*N* ^4^,*N* ^4^-dimethyl­pyridin-4-amine)copper(II)

**DOI:** 10.1107/S1600536809034461

**Published:** 2009-09-05

**Authors:** Yong-Ming Cui, Xi-Bin Dai, Ru-Hua Zha, Qing-Fu Zeng

**Affiliations:** a Engineering Research Center for Clean Production of Textile Dyeing and Printing, Ministry of Education, Wuhan 430073, People’s Republic of China

## Abstract

In the title compound, [Cu(C_8_H_6_BrO_2_)_2_(C_7_H_10_N_2_)_2_(H_2_O)_2_], the Cu^II^ atom (site symmetry 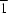) adopts a Jahn–Teller-distorted *trans*-CuN_2_O_4_ octa­hedral coordination, with the aqua O atoms in axially extended sites. An intra­molecular O—H⋯O hydrogen bond helps to establish the conformation and an inter­molecular O—H⋯O hydrogen bond is seen in the crystal packing.

## Related literature

For background to coordination networks, see: Liu & Zhu (2004 ▶); Yang *et al.* (2004 ▶); You *et al.* (2004 ▶). For reference structural data, see: Allen *et al.* (1987 ▶).
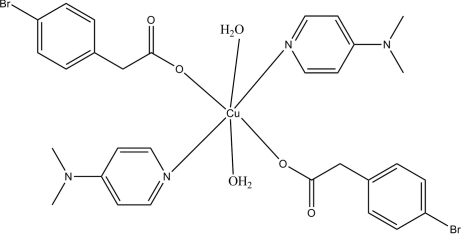


## Experimental

### 

#### Crystal data

[Cu(C_8_H_6_BrO_2_)_2_(C_7_H_10_N_2_)_2_(H_2_O)_2_]
*M* *_r_* = 771.99
Monoclinic, 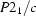

*a* = 10.4792 (10) Å
*b* = 6.1059 (6) Å
*c* = 25.450 (2) Å
β = 100.958 (4)°
*V* = 1598.7 (3) Å^3^
*Z* = 2
Mo *K*α radiation
μ = 3.23 mm^−1^
*T* = 293 K
0.25 × 0.20 × 0.20 mm

#### Data collection

Enraf–Nonius CAD-4 diffractometer
Absorption correction: ψ scan (North *et al.*, 1968 ▶) *T*_min_ = 0.499, *T*_max_ = 0.564
8029 measured reflections
2815 independent reflections
2189 reflections with *I* > 2σ(*I*)
*R*_int_ = 0.026
200 standard reflections every 3 reflections intensity decay: 1%

#### Refinement

*R*[*F* ^2^ > 2σ(*F* ^2^)] = 0.035
*wR*(*F* ^2^) = 0.099
*S* = 1.01
2815 reflections
198 parameters
H-atom parameters constrained
Δρ_max_ = 0.60 e Å^−3^
Δρ_min_ = −0.71 e Å^−3^

### 

Data collection: *CAD-4 Software* (Enraf–Nonius, 1989 ▶); cell refinement: *CAD-4 Software*; data reduction: *XCAD4* (Harms & Wocadlo, 1995 ▶); program(s) used to solve structure: *SHELXS97* (Sheldrick, 2008 ▶); program(s) used to refine structure: *SHELXL97* (Sheldrick, 2008 ▶); molecular graphics: *SHELXTL* (Sheldrick, 2008 ▶); software used to prepare material for publication: *SHELXTL*.

## Supplementary Material

Crystal structure: contains datablocks global, I. DOI: 10.1107/S1600536809034461/hb5065sup1.cif

Structure factors: contains datablocks I. DOI: 10.1107/S1600536809034461/hb5065Isup2.hkl

Additional supplementary materials:  crystallographic information; 3D view; checkCIF report

## Figures and Tables

**Table 1:** Selected bond lengths (Å)

Cu1—O2	2.0006 (17)
Cu1—N2	2.004 (2)
Cu1—O3	2.5052 (19)

**Table 2:** Hydrogen-bond geometry (Å, °)

*D*—H⋯*A*	*D*—H	H⋯*A*	*D*⋯*A*	*D*—H⋯*A*
O3—H3*B*⋯O2^i^	0.90	2.03	2.901 (3)	161
O3—H3*A*⋯O1	0.92	1.79	2.688 (3)	163

Symmetry code: (i) 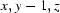.

**Fig. 1.**
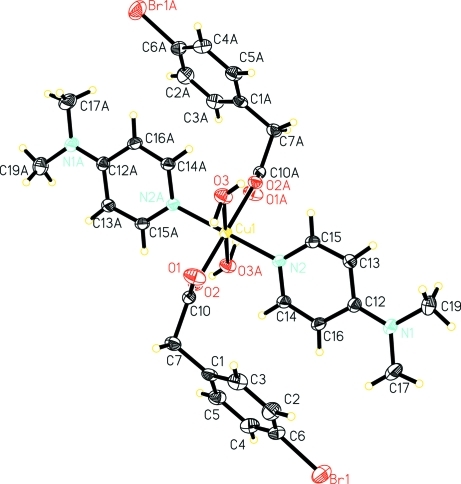
The molecular structure of (I) showing 30% probability displacement ellipsoids. Atoms with the suffix A are generated by the symmetry operation (1–x, 1–y, –z).

## supplementary crystallographic information

### Comment

There has been much research interest in the acid and amine metal complexes due to their molecular architectures (Liu *et al.*, 2004; Yang *et al.*, 2004; You *et al.*, 2004). In this work, we report here the crystal structure of the title compound, (I). In (I), all bond lengths are within normal ranges (Allen *et al.*, 1987) (Fig. 1). The Cu^II^ atom is six-coordinated by two N atoms from *N*,*N*-dimethylpyridin-4-amine, two O atoms from 2-(4-bromophenyl)acetic acid and two O atoms from the water molecules, forming a distorted octahedral coordination.

### Experimental

A mixture of *N*,*N*-dimethylpyridin-4-amine (244 mg, 2 mmol), 2-(4-bromophenyl)acetic acid (428 mg, 2 mmol) and CuCl_2_.2H_2_O (169 mg, 1 mmol) in methanol (10 ml) was stirred for 3 h. After keeping the filtrate in air for 7 d, green blocks of (I) were formed.

### Refinement

All H atoms were positioned geometrically (C—H = 0.93 Å for the aromatic H atoms and C—H = 0.96 Å for the aliphatic H atoms) and were refined as riding, with *U*_iso_(H) = 1.2*U*_eq_(C) and *U*_iso_(H) = 1.2*U*_eq_(N).

### Crystal data

**Table:**

[Cu(C_8_H_6_BrO_2_)_2_(C_7_H_10_N_2_)_2_(H_2_O)_2_]	*F*(000) = 782
*M**_r_* = 771.99	*D*_x_ = 1.604 Mg m^−^^3^
Monoclinic, *P*2_1_/*c*	Mo *K*α radiation, λ = 0.71073 Å
Hall symbol: -P 2ybc	Cell parameters from 25 reflections
*a* = 10.4792 (10) Å	θ = 9–12°
*b* = 6.1059 (6) Å	µ = 3.23 mm^−^^1^
*c* = 25.450 (2) Å	*T* = 293 K
β = 100.958 (4)°	Block, green
*V* = 1598.7 (3) Å^3^	0.25 × 0.20 × 0.20 mm
*Z* = 2	

### Data collection

**Table:**

Enraf–Nonius CAD-4 diffractometer	2189 reflections with *I* > 2σ(*I*)
Radiation source: fine-focus sealed tube	*R*_int_ = 0.026
graphite	θ_max_ = 25.0°, θ_min_ = 1.6°
ω/2θ scans	*h* = −10→12
Absorption correction: ψ scan (North *et al.*, 1968)	*k* = −7→7
*T*_min_ = 0.499, *T*_max_ = 0.564	*l* = −30→28
8029 measured reflections	200 standard reflections every 3 reflections
2815 independent reflections	intensity decay: 1%

### Refinement

**Table:**

Refinement on *F*^2^	Primary atom site location: structure-invariant direct methods
Least-squares matrix: full	Secondary atom site location: difference Fourier map
*R*[*F*^2^ > 2σ(*F*^2^)] = 0.035	Hydrogen site location: inferred from neighbouring sites
*wR*(*F*^2^) = 0.099	H-atom parameters constrained
*S* = 1.01	*w* = 1/[σ^2^(*F*_o_^2^) + (0.0539*P*)^2^ + 0.7463*P*] where *P* = (*F*_o_^2^ + 2*F*_c_^2^)/3
2815 reflections	(Δ/σ)_max_ < 0.001
198 parameters	Δρ_max_ = 0.60 e Å^−^^3^
0 restraints	Δρ_min_ = −0.71 e Å^−^^3^

### Special details

**Table:**

Geometry. All e.s.d.'s (except the e.s.d. in the dihedral angle between two l.s. planes) are estimated using the full covariance matrix. The cell e.s.d.'s are taken into account individually in the estimation of e.s.d.'s in distances, angles and torsion angles; correlations between e.s.d.'s in cell parameters are only used when they are defined by crystal symmetry. An approximate (isotropic) treatment of cell e.s.d.'s is used for estimating e.s.d.'s involving l.s. planes.
Refinement. Refinement of *F*^2^ against ALL reflections. The weighted *R*-factor *wR* and goodness of fit *S* are based on *F*^2^, conventional *R*-factors *R* are based on *F*, with *F* set to zero for negative *F*^2^. The threshold expression of *F*^2^ > σ(*F*^2^) is used only for calculating *R*-factors(gt) *etc*. and is not relevant to the choice of reflections for refinement. *R*-factors based on *F*^2^ are statistically about twice as large as those based on *F*, and *R*- factors based on ALL data will be even larger.

### Fractional atomic coordinates and isotropic or equivalent isotropic displacement parameters (Å^2^)

**Table:**

	*x*	*y*	*z*	*U*_iso_*/*U*_eq_	
Br1	0.10163 (4)	0.98365 (8)	0.229572 (18)	0.0870 (2)	
C1	0.4910 (3)	0.8264 (4)	0.17361 (10)	0.0366 (6)	
C2	0.3209 (3)	0.7210 (6)	0.21980 (12)	0.0567 (9)	
H2	0.2854	0.6195	0.2402	0.068*	
C3	0.4355 (3)	0.6766 (5)	0.20345 (11)	0.0479 (8)	
H3	0.4767	0.5433	0.2126	0.058*	
C4	0.3099 (3)	1.0674 (6)	0.17612 (13)	0.0525 (8)	
H4	0.2669	1.1991	0.1668	0.063*	
C5	0.4259 (3)	1.0225 (5)	0.15993 (13)	0.0462 (8)	
H5	0.4608	1.1250	0.1396	0.055*	
C6	0.2591 (3)	0.9156 (6)	0.20593 (12)	0.0492 (8)	
C7	0.6186 (3)	0.7783 (5)	0.15678 (10)	0.0411 (7)	
H7A	0.6807	0.7252	0.1873	0.049*	
H7B	0.6529	0.9121	0.1443	0.049*	
C10	0.6013 (3)	0.6071 (5)	0.11224 (10)	0.0354 (6)	
C12	0.1011 (3)	0.4106 (5)	0.06736 (10)	0.0354 (6)	
C13	0.1512 (3)	0.2454 (5)	0.03830 (11)	0.0410 (7)	
H13	0.1083	0.1117	0.0324	0.049*	
C14	0.2815 (3)	0.6273 (4)	0.05081 (11)	0.0373 (6)	
H14	0.3254	0.7605	0.0548	0.045*	
C15	0.2618 (3)	0.2798 (5)	0.01872 (11)	0.0392 (7)	
H15	0.2918	0.1658	0.0001	0.047*	
C16	0.1726 (3)	0.6080 (5)	0.07202 (11)	0.0393 (7)	
H16	0.1447	0.7264	0.0899	0.047*	
C17	−0.0511 (4)	0.5523 (6)	0.12039 (17)	0.0694 (11)	
H17A	0.0206	0.6143	0.1450	0.104*	
H17B	−0.1126	0.4918	0.1399	0.104*	
H17C	−0.0924	0.6644	0.0966	0.104*	
C19	−0.0777 (3)	0.1795 (6)	0.08259 (15)	0.0640 (10)	
H19A	−0.1167	0.1622	0.0455	0.096*	
H19B	−0.1445	0.1841	0.1037	0.096*	
H19C	−0.0207	0.0583	0.0939	0.096*	
Cu1	0.5000	0.5000	0.0000	0.03234 (15)	
N1	−0.0042 (2)	0.3814 (4)	0.08968 (10)	0.0462 (6)	
N2	0.3302 (2)	0.4658 (3)	0.02439 (9)	0.0331 (5)	
O1	0.6222 (2)	0.4143 (4)	0.12444 (8)	0.0570 (6)	
O2	0.56545 (17)	0.6809 (3)	0.06509 (7)	0.0372 (4)	
O3	0.58520 (19)	0.1416 (3)	0.03998 (7)	0.0469 (5)	
H3B	0.5591	0.0050	0.0463	0.056*	
H3A	0.6011	0.2105	0.0727	0.056*	

### Atomic displacement parameters (Å^2^)

**Table:**

	*U*^11^	*U*^22^	*U*^33^	*U*^12^	*U*^13^	*U*^23^
Br1	0.0564 (3)	0.1343 (5)	0.0770 (3)	−0.0039 (2)	0.0294 (2)	−0.0350 (3)
C1	0.0497 (17)	0.0354 (15)	0.0256 (13)	−0.0051 (13)	0.0092 (12)	−0.0073 (11)
C2	0.069 (2)	0.065 (2)	0.0416 (17)	−0.0128 (19)	0.0260 (16)	0.0034 (15)
C3	0.065 (2)	0.0413 (17)	0.0386 (15)	0.0014 (16)	0.0123 (15)	0.0054 (13)
C4	0.061 (2)	0.0482 (18)	0.0505 (18)	0.0102 (17)	0.0149 (16)	−0.0055 (15)
C5	0.060 (2)	0.0394 (18)	0.0421 (16)	−0.0025 (15)	0.0167 (15)	−0.0010 (13)
C6	0.0488 (18)	0.064 (2)	0.0388 (16)	−0.0027 (17)	0.0171 (14)	−0.0152 (15)
C7	0.0470 (17)	0.0440 (17)	0.0323 (14)	−0.0046 (14)	0.0080 (12)	−0.0083 (12)
C10	0.0345 (15)	0.0392 (17)	0.0358 (15)	−0.0073 (13)	0.0155 (12)	−0.0071 (12)
C12	0.0330 (14)	0.0396 (15)	0.0348 (14)	−0.0011 (13)	0.0093 (12)	0.0013 (12)
C13	0.0413 (16)	0.0349 (15)	0.0505 (16)	−0.0085 (13)	0.0177 (13)	−0.0096 (13)
C14	0.0407 (15)	0.0298 (15)	0.0447 (16)	−0.0029 (12)	0.0164 (13)	−0.0063 (12)
C15	0.0437 (16)	0.0334 (15)	0.0452 (16)	−0.0045 (13)	0.0199 (13)	−0.0103 (12)
C16	0.0405 (16)	0.0337 (16)	0.0477 (16)	0.0018 (13)	0.0188 (13)	−0.0063 (13)
C17	0.062 (2)	0.073 (2)	0.087 (3)	−0.0086 (19)	0.048 (2)	−0.017 (2)
C19	0.054 (2)	0.067 (2)	0.079 (2)	−0.0204 (18)	0.0334 (18)	−0.0083 (19)
Cu1	0.0321 (3)	0.0372 (3)	0.0304 (3)	−0.0056 (2)	0.01259 (19)	−0.00735 (18)
N1	0.0408 (14)	0.0468 (15)	0.0572 (15)	−0.0077 (12)	0.0249 (12)	−0.0071 (12)
N2	0.0355 (12)	0.0318 (13)	0.0351 (12)	−0.0014 (10)	0.0143 (10)	−0.0048 (9)
O1	0.0861 (18)	0.0371 (12)	0.0473 (12)	−0.0003 (12)	0.0116 (11)	−0.0025 (10)
O2	0.0436 (11)	0.0381 (11)	0.0317 (10)	−0.0060 (9)	0.0114 (8)	−0.0075 (8)
O3	0.0602 (13)	0.0386 (11)	0.0438 (11)	−0.0036 (10)	0.0146 (9)	−0.0017 (9)

### Geometric parameters (Å, °)

**Table:**

Br1—C6	1.906 (3)	C14—N2	1.347 (3)
C1—C3	1.386 (4)	C14—C16	1.357 (4)
C1—C5	1.389 (4)	C14—H14	0.9300
C1—C7	1.508 (4)	C15—N2	1.336 (3)
C2—C6	1.367 (5)	C15—H15	0.9300
C2—C3	1.371 (4)	C16—H16	0.9300
C2—H2	0.9300	C17—N1	1.445 (4)
C3—H3	0.9300	C17—H17A	0.9600
C4—C6	1.368 (5)	C17—H17B	0.9600
C4—C5	1.384 (5)	C17—H17C	0.9600
C4—H4	0.9300	C19—N1	1.447 (4)
C5—H5	0.9300	C19—H19A	0.9600
C7—C10	1.527 (4)	C19—H19B	0.9600
C7—H7A	0.9700	C19—H19C	0.9600
C7—H7B	0.9700	Cu1—O2^i^	2.0006 (17)
C10—O1	1.226 (4)	Cu1—O2	2.0006 (17)
C10—O2	1.270 (3)	Cu1—N2	2.004 (2)
C12—N1	1.345 (3)	Cu1—N2^i^	2.004 (2)
C12—C13	1.410 (4)	Cu1—O3	2.5052 (19)
C12—C16	1.412 (4)	Cu1—O3^i^	2.5052 (19)
C13—C15	1.362 (4)	O3—H3B	0.9018
C13—H13	0.9300	O3—H3A	0.9200
			
C3—C1—C5	117.9 (3)	C14—C16—C12	120.9 (3)
C3—C1—C7	121.0 (3)	C14—C16—H16	119.6
C5—C1—C7	121.1 (3)	C12—C16—H16	119.6
C6—C2—C3	119.5 (3)	N1—C17—H17A	109.5
C6—C2—H2	120.2	N1—C17—H17B	109.5
C3—C2—H2	120.2	H17A—C17—H17B	109.5
C2—C3—C1	121.3 (3)	N1—C17—H17C	109.5
C2—C3—H3	119.4	H17A—C17—H17C	109.5
C1—C3—H3	119.4	H17B—C17—H17C	109.5
C6—C4—C5	119.2 (3)	N1—C19—H19A	109.5
C6—C4—H4	120.4	N1—C19—H19B	109.5
C5—C4—H4	120.4	H19A—C19—H19B	109.5
C4—C5—C1	120.9 (3)	N1—C19—H19C	109.5
C4—C5—H5	119.5	H19A—C19—H19C	109.5
C1—C5—H5	119.5	H19B—C19—H19C	109.5
C2—C6—C4	121.1 (3)	O2^i^—Cu1—O2	180.00 (6)
C2—C6—Br1	120.3 (3)	O2^i^—Cu1—N2	90.81 (8)
C4—C6—Br1	118.6 (3)	O2—Cu1—N2	89.19 (8)
C1—C7—C10	111.0 (2)	O2^i^—Cu1—N2^i^	89.19 (8)
C1—C7—H7A	109.4	O2—Cu1—N2^i^	90.81 (8)
C10—C7—H7A	109.4	N2—Cu1—N2^i^	180.00 (11)
C1—C7—H7B	109.4	O2—Cu1—O3	96.11 (7)
C10—C7—H7B	109.4	O2—Cu1—O3^i^	83.89 (7)
H7A—C7—H7B	108.0	O3—Cu1—N2	92.98 (7)
O1—C10—O2	125.9 (2)	O3—Cu1—O2^i^	83.89 (7)
O1—C10—C7	118.6 (2)	O3—Cu1—O3^i^	180.00 (7)
O2—C10—C7	115.5 (2)	O3—Cu1—N2^i^	87.02 (7)
N1—C12—C13	122.9 (3)	N2—Cu1—O3^i^	87.02 (7)
N1—C12—C16	122.8 (3)	O2^i^—Cu1—O3^i^	96.11 (7)
C13—C12—C16	114.2 (2)	O3^i^—Cu1—N2^i^	92.98 (7)
C15—C13—C12	120.6 (3)	C12—N1—C17	121.5 (3)
C15—C13—H13	119.7	C12—N1—C19	121.4 (3)
C12—C13—H13	119.7	C17—N1—C19	117.1 (3)
N2—C14—C16	124.2 (3)	C15—N2—C14	115.4 (2)
N2—C14—H14	117.9	C15—N2—Cu1	123.04 (18)
C16—C14—H14	117.9	C14—N2—Cu1	121.40 (18)
N2—C15—C13	124.6 (2)	C10—O2—Cu1	125.44 (18)
N2—C15—H15	117.7	H3B—O3—H3A	105.6
C13—C15—H15	117.7		

Symmetry codes: (i) −*x*+1, −*y*+1, −*z*.

### Hydrogen-bond geometry (Å, °)

**Table:**

*D*—H···*A*	*D*—H	H···*A*	*D*···*A*	*D*—H···*A*
O3—H3B···O2^ii^	0.90	2.03	2.901 (3)	161
O3—H3A···O1	0.92	1.79	2.688 (3)	163

Symmetry codes: (ii) *x*, *y*−1, *z*.
